# Supplementary material for: Orchestrating and sharing large multimodal data for transparent and reproducible research
Source: Nat Commun. 2021 Oct 4;12:5797. doi: 10.1038/s41467-021-25974-w (PMC8490371; doi:10.1038/s41467-021-25974-w)
Supplement: Supplementary file 1 — Supplementary Information [file 41467_2021_25974_MOESM1_ESM.pdf]

SUPPLEMENTARY INFORMATION

Orchestrating and sharing large multimodal data for transparent and reproducible research

Supplementary Methods

All analyses can be reproduced through our custom Code Ocean compute capsule: https://codeocean.com/capsule/9215268/tree

Pharmacogenomics

In order to identify an association between ERBB2 gene expression and lapatinib drug response across pharmacogenomic datasets (predictive biomarker) [1] , log_2 normalized gene expression data computed by Kallisto was obtained from the following data objects: GRAY (https://orcestra.ca/pset/10.5281/zenodo.4557735), UHNBreast (https://orcestra.ca/pset/10.5281/zenodo.3905460), CCLE (https://orcestra.ca/pset/10.5281/zenodo.3905462), and GDSC2 (https://orcestra.ca/pset/10.5281/zenodo.3905481). Drug response data was also obtained from the same data objects, in the form of AAC (Area-above the drug dose response curve). The concordance index was then computed between gene expression and lapatinib drug response for each dataset using the *survcomp* Bioconductor package (v.1.42.0), as well as in the form of a meta-analysis (N=1,281) to identify ERBB2's predictive value across all datasets. In order to identify the consistency of lapatinib drug response (AAC) across pharmacogenomic datasets the Pearson correlation coefficient was computed between CTRPv2/GDSC 1 (N=511) and CTRPv2/GDSC2 (N=587). GDSC1 and GDSC2 utilize varying pharmacological assays for their drug response data, impacting the correlation with CTRPv2 and use of the data for biomarker discovery.

Toxicogenomics

To highlight the top differentially expressed genes for "most drug induced liver injury" (DILI) drug acetaminophen and "no DILI" drug chloramphenicol in Open TG-GATEs Human data (https://orcestra.ca/toxicoset/10.5281/zenodo.4302218), the data object was downloaded from ORCESTRA [2]. Differential gene expression for both drugs were analyzed using *limma* pipeline in the *computeLimmaDiffExpr* function from ToxicoGx Bioconductor package (v.1.2.1). Differentially expressed genes for dose "High" at 24 hours were filtered. 6575 genes examined for acetaminophen, while 895 genes were examined for chloramphenicol. To rank genes in the order of evidence for differential expression analyses, an empirical Bayes moderated t-statistics was used for each individual contrast equal to zero. Then, the two-sided p-values corresponding to the t-statistics were corrected for multiple comparisons using Benjamini & Hochberg method to control false discovery rate (FDR).

Xenographic Pharmacogenomics

To investigate the correlation between ERBB2 expression and trastuzumab drug response across breast cancer patient-derived xenograft models (N=37) [3], the PDXE data object was utilized (https://orcestra.ca/xevaset/10.5281/zenodo.4302463), which was generated with the Xeva Bioconductor package (v.1.8.0). ERBB2 log_2 normalized

gene expression data computed by Kallisto 0.46.1 was obtained from the data object, along with trastuzumab drug response in the form of AAC (area-above the tumour growth curve). The Pearson correlation coefficient was computed between gene expression and drug response, in order to identify the potential correlation.

Clinical Genomics

Patient risk across pancreatic cancer patients (N=1102) was investigated through identifying the prognostic value of a Pancreatic Cancer Overall Survival Predictor (PCOSP) model, which implements an novel ensemble of gene expression binary k-top scoring pair classifiers (kTSP). The MetaGxPancreas data object was utilized on ORCESTRA (https://orcestra.ca/clinicalgenomics/10.5281/zenodo.4312144). The model produces a PCOSP score, allowing for the estimation of patient risk. To benchmark this method, we have compared prediction performance against a standard linear model of clinical parameters sex, age, and TNM status using concordance index. The cohort was split into training and testing sets, where the PCOSP model was fit using the PCOSP function in the *PDATK* R Bioconductor Package, while comparing with an ensemble of kTSP classifiers were then trained, in order to predict patient risk. A generalized linear model (GLM) was fit based on clinical parameters to benchmark PCOSP model, where the ClinicalModel vs PCOSP model performance was compared using the *compareModels* function in PDATK.

Radiogenomics

To investigate the association between pathway-level features and radiosensitivity, we used the ORCESTRA Radiogenomics data object (https://orcestra.ca/radioset/10.5281/zenodo.4313029), which contains a panel of 540 cancer cell lines treated with gamma radiation from 137Cs at eight doses, up to 10 Gy. To summarize the sensitivity of the cell lines to radiation, we fit a linear-quadratic curve to the dose response data, and computed the area under the fitted radiation survival curve (AUC). Large values of AUC indicate resistance to radiation, while small values indicate sensitivity. Next, we extracted the RNA-Seq gene expression data from the RadioSet, which were quantified with Kallisto 0.46.1 and calculated as log2 of TPM. To identify genes relevant to radiosensitivity, we computed the Pearson correlation between gene expression and AUC controlling for tissue in both the expression and radiosensitivity values.

Supplementary Tables

Category	Dataset name	No. of samples	No. of perturbations	Release date/version
Pharmacogenomics (in vitro)	GRAY[4]	73	89	2013
	GRAY[5]	74	107	2017
	FIMM[6]	50	52	2016
	GDSC1[7]	1104	343	2020(v1-8.2)
	GDSC2[8]	1104	190	2020(v2-8.2)
	gCSI[9]	747	16	2017
	UHNBreast[10]	84	8	2019
	CTRPv2[11]	887	544	2015
	CCLE[12]	1094	24	2015
	GDSC1[13]	1104	303	2019(v1-8.0)
	GDSC2[14]	1104	169	2019(v2-8.0)
Pharmacogenomics (in vivo)	PDXE[15]	277	62	2015
Radiogenomics	Cleveland[16]	540	1	2016
Toxicogenomics	DrugMatrix[17]	1	126	2019
	EMEXP2458[18]	2	6	2010
	Open TG-GATEs[19]	2	152	2015
Clinical Genomics	MetaGxPancreas[20]	1792	NA	2019

Supplementary Table 1. Summary of data types with respective dataset names, samples, perturbations, and releases available through ORCESTRA.

Data Portal	Notable Functionalities	Expected Functionalities
SYNAPSE[21]	Wiki integration<br>Data provenance tracker<br>Dataset discussion board	Track updates to a dataset at the file level
Broad Institute (CCLE)[22]	Easy access of current, previous, and legacy data versions<br>Display of data by data-type<br>(e.g. pharmacological profiling, mRNA expression)	Display of data processing tools, and their versions, with all accompanying files (e.g. transcriptome)
DRYAD[23]	Simplistic navigation<br>Dataset metrics<br>Respective publication download	Direct access to all processing pipelines utilized
NCBI[24]	SRA sequencing information with<br>additional sample metadata	Option of downloading processed data through multiple methods/pipelines
CancerRxGene (GDSC)[25]	Direct integration of cell line and drug metadata<br>Direct links to raw data hosted on other portals<br>Description of changes to each drug sensitivity data release	

Supplementary Table 2. Common data portals for sharing genomics data with notable and expected functionalities for transparent and reproducible processing, analysis, and interpretation.

Supplementary Figures

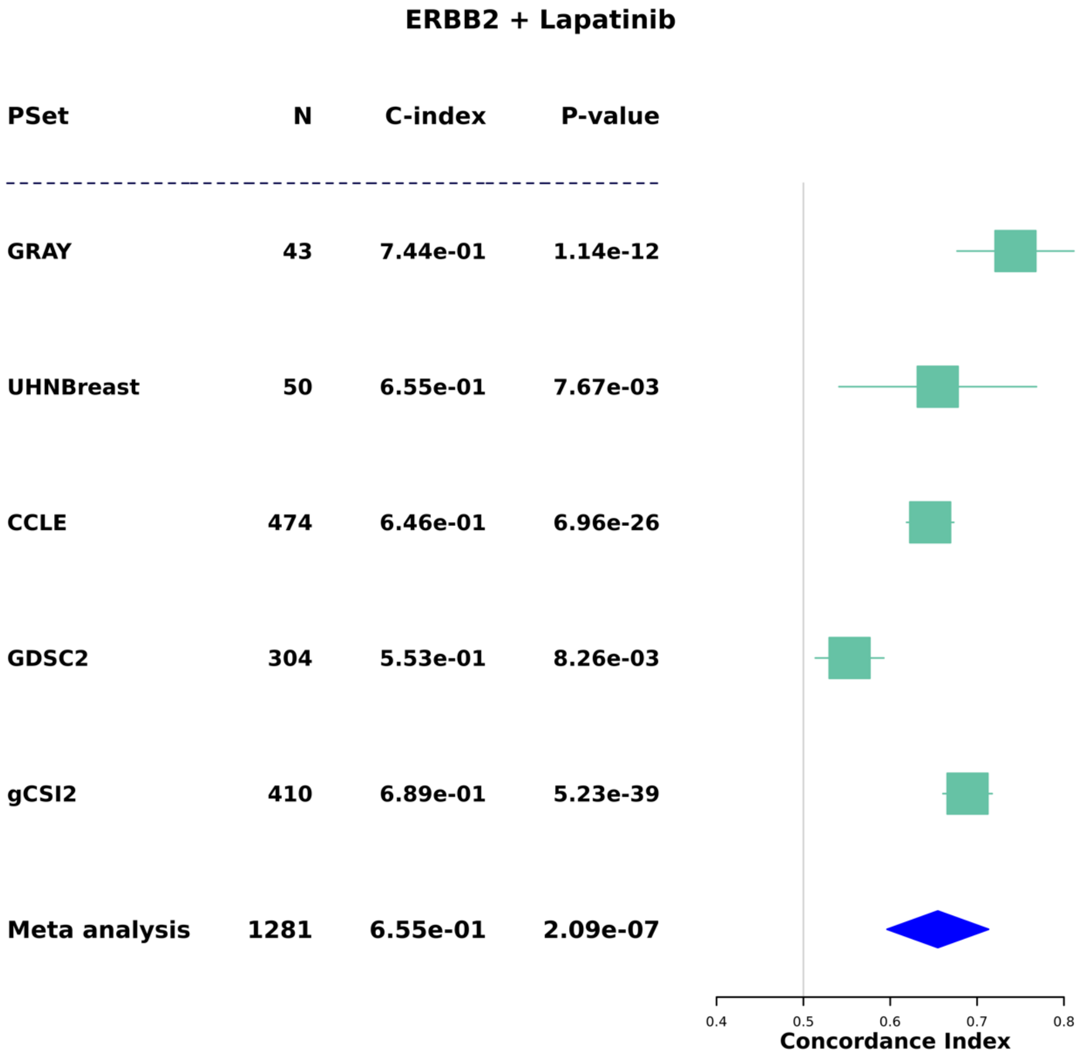

Supplementary Fig. 1. Concordance of ERBB2 expression and Lapatinib drug response across GRAY, UHNBreast, CCLE, GDSC2, and gCSI2 data objects generated by ORCESTRA. PSet: PharmacoSet; C-index: concordance-index; *N*: number of cell lines. Meta analysis represents combined concordance index and p-value across PSets. n=1281 cancer cell lines. 95% confidence interval displayed for each PSet and meta analysis. A two-sided alternative hypothesis was selected when computing the concordance index.

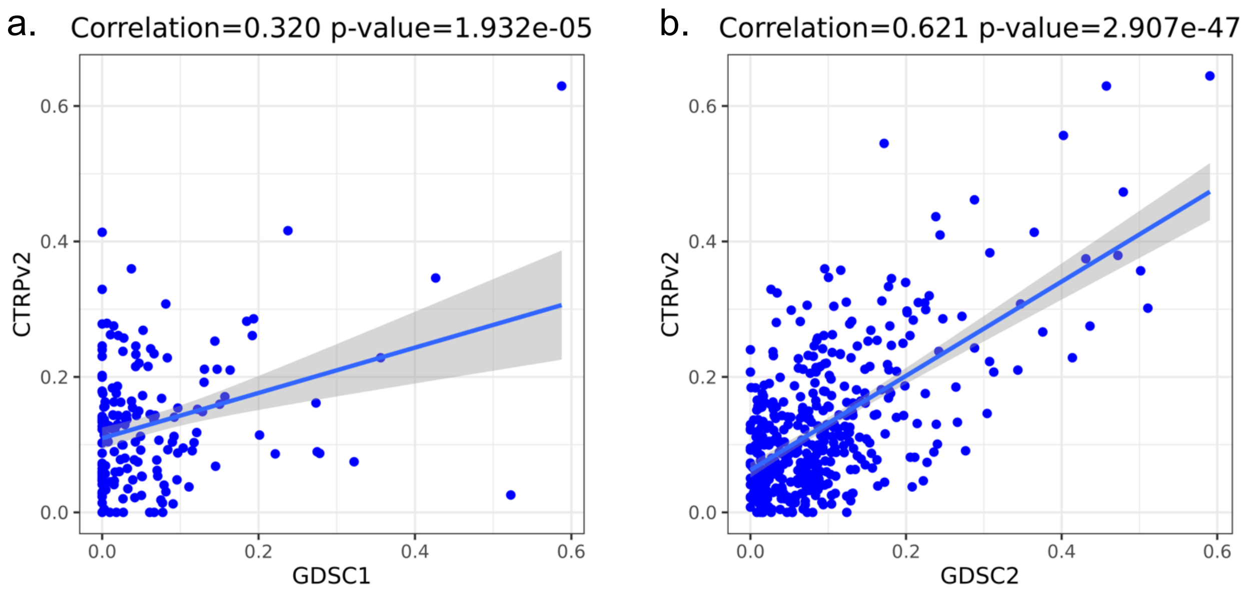

Supplementary Fig. 2. Pearson correlation between GDSC1/2 and CTRPv2 Lapatinib drug response. **a.** GDSC1 vs CTRPv2. **b.** GDSC2 vs CTRPv2. *N*=587 cell lines intersected between GDSC1 and CTRPv2, while 511 cell lines intersected between GDSC2 and CTRPv2. The error bands represent a 95% confidence interval. A two-sided Pearson correlation test was performed.

a.

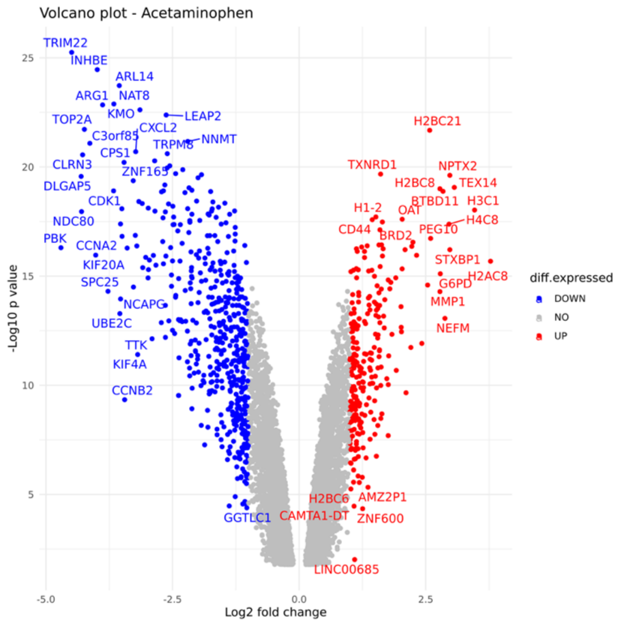

b.

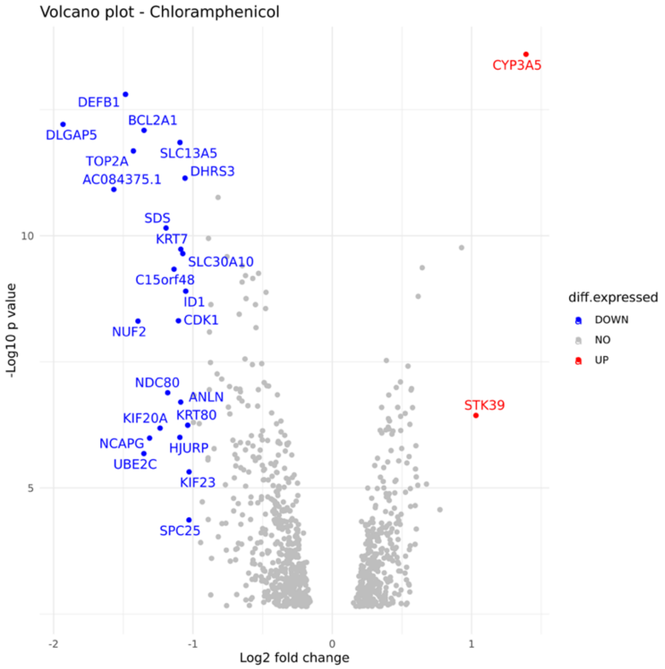

Supplementary Fig. 3. Differential gene expression analysis of acetaminophen and chloramphenicol. Genes with log2 fold change > 1 and p value < 0.05 are highlighted as Up-regulated genes (red) and genes with log2 fold change < -1 and p value < 0.05 are highlighted as Down-regulated genes (blue) for acetaminophen (**a**) and chloramphenicol (**b**). The genes excluded from the above filter are shown in grey. 6575 genes examined for acetaminophen, while 895 genes were examined for chloramphenicol. To rank genes in the order of evidence for differential expression analyses, an empirical Bayes moderated t-statistics was used. Two-sided p-values corresponding to the t-statistics were corrected for multiple comparisons using Benjamini & Hochberg method to control false discovery rate (FDR).

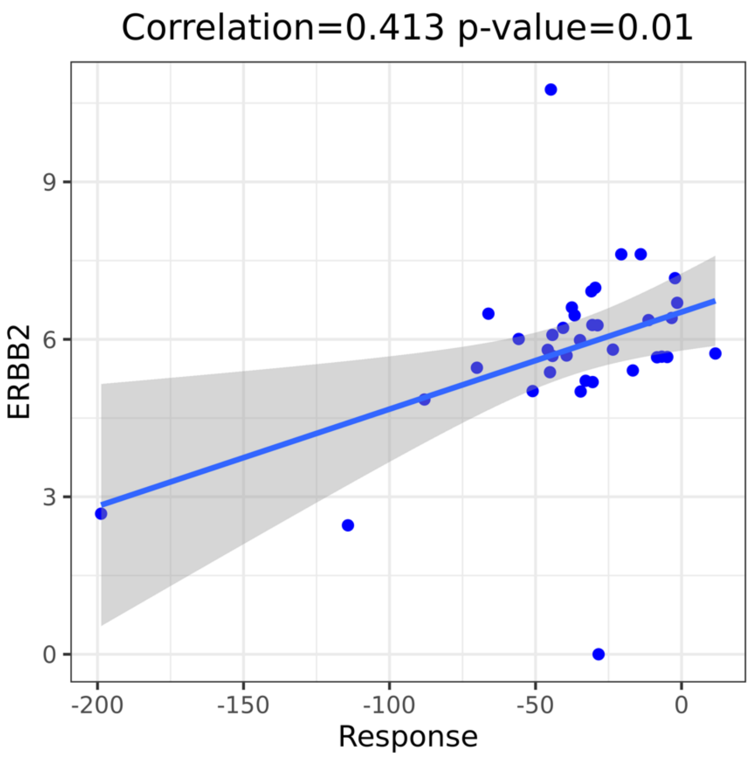

Supplementary Fig. 4. Correlation of ERBB2 expression and trastuzumab drug response. The x-axis represents the best average response of trastuzumab breast cancer patient derived xenograft models. The y-axis displays ERBB2 gene expression. N=37 samples. The error band represents a 95% confidence interval. A two-sided Pearson correlation test was performed.

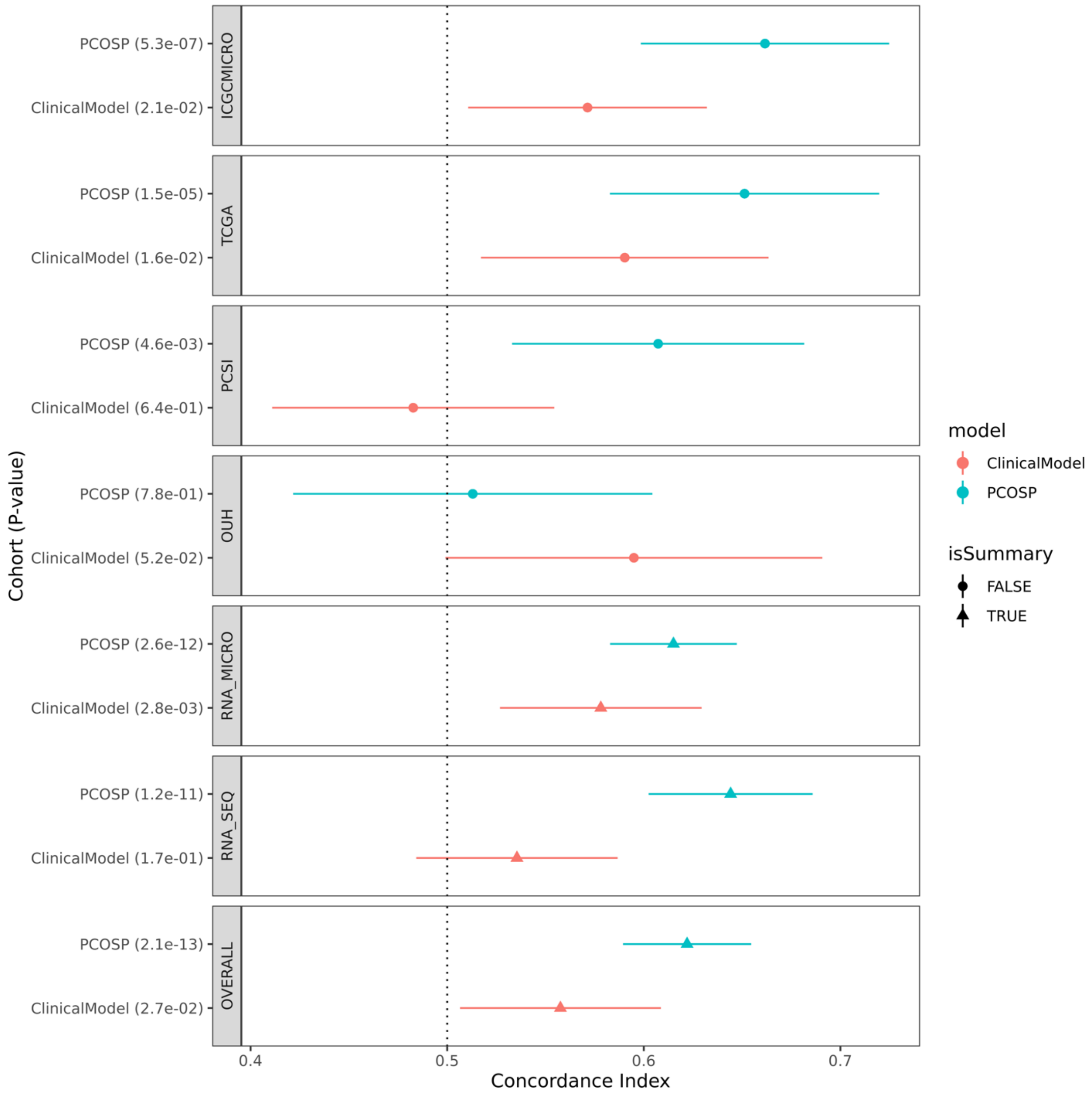

Supplementary Fig. 5. Comparison of concordance index of patient prognosis classification between PCOSP and clinical models. The Pancreatic Cancer Overall Survival Predictor (PCOSP) model implements a novel ensemble of gene expression binary k-top scoring pair classifiers to produce a PCOSP score estimating patient risk. PCOSP score is defined as the proportion of classifiers which predict prognosis as good, with a score of less than 0.5 indicating poor prognosis. To benchmark this method, prediction performance is compared against a standard linear model of clinical parameters sex, age, and TNM status using concordance index. 95% confidence interval displayed for each cohort. *N*=1102 patient samples. The measure of centre for the error bars is the mean, computed through the survcomp::combine.est function via the survcomp Bioconductor package.

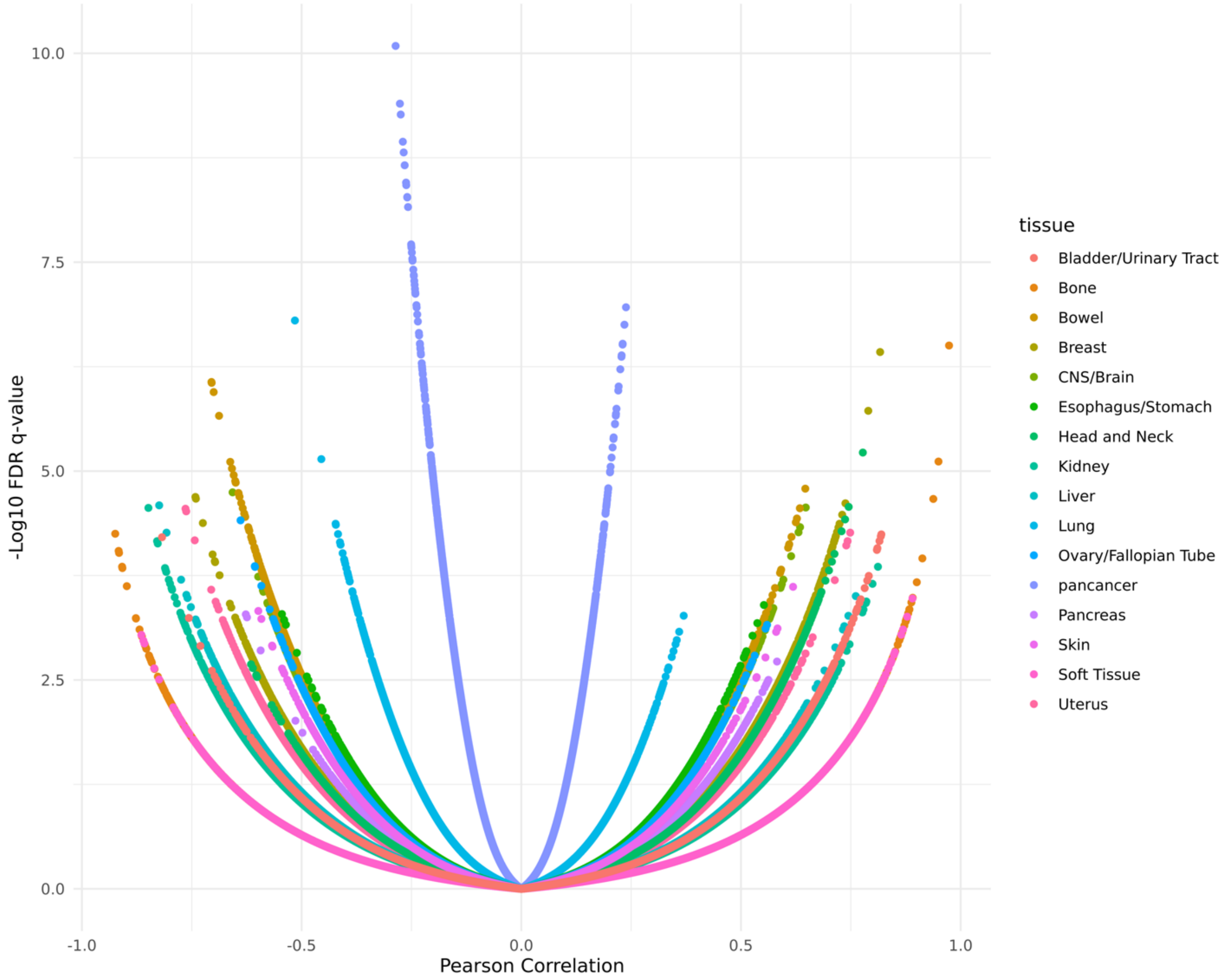

Supplementary Fig. 6. The waterfall plot depicts the Pearson correlation (x-axis) between individual genes and radiosensitivity (AUC). Each point represents one gene, and each color is a different set of cell lines, grouped by tissue. The -log10 FDR-adjusted p-values from the two-tailed Pearson correlation test are displayed on the y-axis. RNA-sequencing gene expression data are log2 of TPM, while radiosensitivity is the area under the fitted radiation survival curve (AUC). *N*=540 cancer cell lines.

Supplementary References

1. Mammoliti, A., Smirnov, P., Safikhani, Z., Ba-Alawi, W. & Haibe-Kains, B. Creating reproducible pharmacogenomic analysis pipelines. *Sci Data* **6**, 166 (2019).
2. Nair, S. K. *et al.* ToxicoDB: an integrated database to mine and visualize large-scale toxicogenomic datasets. *Nucleic Acids Res.* **48**, W455–W462 (2020).
3. Mer, A. S. *et al.* Integrative Pharmacogenomics Analysis of Patient-Derived Xenografts. *Cancer Res.* **79**, 4539–4550 (2019).
4. Haibe-Kains, B. GRAY. (2020) doi:10.5281/ZENODO.3905454.
5. Haibe-Kains, B. GRAY2017. (2021) doi:10.5281/ZENODO.4557735.
6. Haibe-Kains, B. FIMM. (2020) doi:10.5281/ZENODO.3905448.
7. Haibe-Kains, B. GDSC1. (2020) doi:10.5281/ZENODO.3905485.
8. Haibe-Kains, B. GDSC2. (2020) doi:10.5281/ZENODO.4012486.
9. Haibe-Kains, B. gCSI. (2021) doi:10.5281/ZENODO.4742696.
10. Haibe-Kains, B. UHNBreast. (2020) doi:10.5281/ZENODO.3905460.
11. Haibe-Kains, B. CTRPv2. (2020) doi:10.5281/ZENODO.3905470.
12. Haibe-Kains, B. CCLE. (2020) doi:10.5281/ZENODO.3905462.
13. Haibe-Kains, B. GDSC1-8.0. (2020) doi:10.5281/zenodo.3905505.
14. Haibe-Kains, B. GDSC2-8.0. (2020) doi:10.5281/ZENODO.4012486.
15. Haibe-Kains, B. Xeva_PDXE. (2020) doi:10.5281/ZENODO.4302463.
16. Haibe-Kains, B. Cleveland. (2020) doi:10.5281/ZENODO.4313029.
17. Haibe-Kains, B. drugMatrix. (2020) doi:10.5281/ZENODO.4302202.
18. Haibe-Kains, B. EMEXP2458. (2020) doi:10.5281/ZENODO.4302212.
19. Haibe-Kains, B. TGGATES_human_ldh. (2020) doi:10.5281/ZENODO.4302218.
20. Haibe-Kains, B. MetaGxPancreas. (2020) doi:10.5281/ZENODO.4312144.
21. Bionetworks, S. Synapse. *Synapse* https://www.synapse.org/.

22. Broad Institute Data Portal. https://portals.broadinstitute.org/ccle/data.

23. Dryad - Publish and Preserve your Data. *Dryad* https://datadryad.org/stash.

24. NCBI - National Center for Biotechnology Information. *National Center for Biotechnology Information* https://www.ncbi.nlm.nih.gov/.

25. Cancerrxgene - genomics of drug sensitivity in cancer. *Genomics of Drug Sensitivity in Cancer* https://www.cancerrxgene.org/.
