# Supplementary material for: Orchestrating and sharing large multimodal data for transparent and reproducible research
Source: Nat Commun. 2021 Oct 4;12:5797. doi: 10.1038/s41467-021-25974-w (PMC8490371; doi:10.1038/s41467-021-25974-w)
Supplement: Supplementary file 2 — Reporting Summary [file 41467_2021_25974_MOESM2_ESM.pdf]

nature research

Corresponding author(s): Dr. Benjamin Haibe-Kains

Last updated by author(s): Aug 12, 2021

Reporting Summary

Nature Research wishes to improve the reproducibility of the work that we publish. This form provides structure for consistency and transparency in reporting. For further information on Nature Research policies, see our Editorial Policies and the Editorial Policy Checklist.

Statistics

For all statistical analyses, confirm that the following items are present in the figure legend, table legend, main text, or Methods section.

n/a	Confirmed	
☐	☒	The exact sample size (n) for each experimental group/condition, given as a discrete number and unit of measurement
☒	☐	A statement on whether measurements were taken from distinct samples or whether the same sample was measured repeatedly
☐	☒	The statistical test(s) used AND whether they are one- or two-sided<br>*Only common tests should be described solely by name; describe more complex techniques in the Methods section.*
☒	☐	A description of all covariates tested
☒	☐	A description of any assumptions or corrections, such as tests of normality and adjustment for multiple comparisons
☐	☒	A full description of the statistical parameters including central tendency (e.g. means) or other basic estimates (e.g. regression coefficient) AND variation (e.g. standard deviation) or associated estimates of uncertainty (e.g. confidence intervals)
☐	☒	For null hypothesis testing, the test statistic (e.g. F, t, r) with confidence intervals, effect sizes, degrees of freedom and P value noted<br>*Give P values as exact values whenever suitable.*
☒	☐	For Bayesian analysis, information on the choice of priors and Markov chain Monte Carlo settings
☒	☐	For hierarchical and complex designs, identification of the appropriate level for tests and full reporting of outcomes
☐	☒	Estimates of effect sizes (e.g. Cohen's d, Pearson's r), indicating how they were calculated

Our web collection on statistics for biologists contains articles on many of the points above.

Software and code

Policy information about availability of computer code

Data collection	No software was used for data collection.
Data analysis	Software/tools used for analysis: 1) Pachyderm tool (v.1.9.3); 2) PharmacoGx R package (v.2.4.0); 3) ToxicoGx R package (1.2.1); 4) Xeva R package (v.1.8.0); MetaGxPancreas R package (v.1.12.1); RadioGx R package (v.1.2.0); Kallisto tool (v.0.43.0, 0.46.1); Salmon tool (v. 0.8.2, 1.1.0).<br><br>All code for data object generation in ORCESTRA is publicly available on GitHub: https://github.com/BHKLAB-Pachyderm. Code for the case studies in the manuscript can be accessed in a custom compute capsule on Code Ocean here: https://codeocean.com/capsule/9215268/tree

For manuscripts utilizing custom algorithms or software that are central to the research but not yet described in published literature, software must be made available to editors and reviewers. We strongly encourage code deposition in a community repository (e.g. GitHub). See the Nature Research guidelines for submitting code & software for further information.

Data

Policy information about availability of data

All manuscripts must include a data availability statement. This statement should provide the following information, where applicable:

- Accession codes, unique identifiers, or web links for publicly available datasets
- A list of figures that have associated raw data
- A description of any restrictions on data availability

Databases used in study:
1) GRAY dataset (https://datadryad.org/stash/dataset/doi:10.5061/dryad.03n60): The data are provided under CC0 1.0 Universal (CC0 1.0) Public Domain Dedication license.

2) CCLE dataset (https://data.broadinstitute.org/ccle_legacy_data): CCLE data, are provided under Creative Commons Attribution 4.0 license.
3) CTRPv2 dataset (https://portals.broadinstitute.org/ctrp): CTRPv2 data, are provided under Creative Commons Attribution 4.0 license.
4) gCSI dataset (http://research-pub.gene.com/gCSI_GRvalues2019/): gCSI data are provided under Creative Commons BY 4.0 license.
5) FIMM dataset (https://doi.org/10.1038/nature20171): FIMM data are provided under Creative Commons BY 4.0 license.
6) GDSC dataset (https://www.cancerrxgene.org): GDSC data have the following data usage policy: https://depmap.sanger.ac.uk/documentation/data-usage-policy/
7) UHNBreast dataset (https://codeocean.com/capsule/6718332/). UHNBreast data are provided under Creative Commons BY 4.0 license.
8) Open TG-GATEs dataset (Lifescience Database Archive- https://dbarchive.biosciencedbc.jp/en/open-tggates/download.html): Open TG-GATEs data are provided under Creative Commons Attribution-Share Alike 2.1 Japan.
9) EMEXP2458 dataset (https://www.ebi.ac.uk/arrayexpress/experiments/E-MEXP-2458/): EMEXP2458 data are provided under permissive license: https://www.ebi.ac.uk/arrayexpress/help/FAQ.html#data_restrictions
10) DrugMatrix dataset (diXa Data Warehouse - wwwdev.ebi.ac.uk/fg/dixa/ - study ID DIXA-033). The DrugMatrix dataset is attributed to the National Toxicology Program and may be copied and distributed without permission.
11) PDXE dataset (https://pubmed.ncbi.nlm.nih.gov/26479923/). The PDXE dataset may be utilized under NCBI and author guidelines.
12) Cleveland dataset (https://ctd2.nci.nih.gov/dataPortal/). The Cleveland dataset data is in accordance to the CTD2 policy here: https://ocg.cancer.gov/programs/ctd2/using-ctd2-data
13) MetaGxPancreas dataset (http://bioconductor.org/packages/release/data/experiment/html/MetaGxPancreas.html). The MetaGxPancreas data is under Creative Commons Attribution 4.0 International License
All of the data are publicly available on ORCESTRA (orcestra.ca) via dedicated documented
webpages, which include respective digital object identifiers (DOI) and Zenodo links for each data object generated. Data for the case studies in the manuscript can be accessed in a custom compute capsule on Code Ocean here: https://codeocean.com/capsule/9215268/tree

Field-specific reporting

Please select the one below that is the best fit for your research. If you are not sure, read the appropriate sections before making your selection.

☒ Life sciences ☐ Behavioural & social sciences ☐ Ecological, evolutionary & environmental sciences

For a reference copy of the document with all sections, see nature.com/documents/nr-reporting-summary-flat.pdf

Life sciences study design

All studies must disclose on these points even when the disclosure is negative.

Sample size	17 datasets were utilized by our ORCESTRA platform to showcase its flexibility in integrating data from various modalities. The number of datasets involved in the study were determined by identifying biomedical datasets that possess sufficient data for biomarker discovery from the following datatypes: pharmacogenomics, xenographic pharmacogenomics, toxicogenomics, clinical genomics, and radiogenomics. These datasets are sufficient for the analysis, as they allow users to identify potential biomarkers predictive of drug response, as demonstrated in the Supplementary Info.
Data exclusions	No data were excluded in our manuscript.
Replication	All data objects generated by our platform can be reproduced, as (1) All data is publicly available on ORCESTRA (orcestra.ca); (2) All code, including the reproducible pipelines for data object generation, is publicly available on GitHub: https://github.com/BHKLAB-Pachyderm; (3) All data objects are assigned a Zenodo DOI on orcestra.ca.
Randomization	Randomization is not relevant for our manuscript. Each of the 17 datasets utilized by our ORCESTRA platform contain samples unique to their respective dataset.
Blinding	Blinding is not relevant for our manuscript, as all 17 datasets utilized by our platform were publicly available and were therefore not concealed.

Reporting for specific materials, systems and methods

We require information from authors about some types of materials, experimental systems and methods used in many studies. Here, indicate whether each material, system or method listed is relevant to your study. If you are not sure if a list item applies to your research, read the appropriate section before selecting a response.

Materials & experimental systems

n/a	Involved in the study
☒	☐ Antibodies
☒	☐ Eukaryotic cell lines
☒	☐ Palaeontology and archaeology
☒	☐ Animals and other organisms
☒	☐ Human research participants
☒	☐ Clinical data
☒	☐ Dual use research of concern

Methods

n/a	Involved in the study
☒	☐ ChIP-seq
☒	☐ Flow cytometry
☒	☐ MRI-based neuroimaging
